# A pedagogical study on promoting students' deep learning through design-based learning

**DOI:** 10.1007/s10798-022-09789-4

**Published:** 2022-11-27

**Authors:** Chunmeng Weng, Congying Chen, Xianfeng Ai

**Affiliations:** School of Art and Design, Wuhan University of Science and Technology, Wuhan, 430065 China

**Keywords:** Design-based learning, Deep learning, Teaching evaluation, Individual competency

## Abstract

This paper illustrates the design-based learning (DBL) approach to promoting the deep learning of students and improving the quality of teaching in engineering design education. We performed three aspects of research with students in a typical educational activity. The first study investigated students' deep learning before and after the DBL approach, both in terms of deep learning status and deep learning ability. The second study examined the effectiveness of the DBL approach by comparative research of a control class (traditional teaching method) and an experimental class (DBL method). The third study examined students' evaluations of the DBL approach. It is approved that the DBL approach has distinctively stimulated the students' motivation to learn, making them more actively engaged in study. The students' higher-order thinking and higher-order capabilities are enhanced, such as critical thinking ability and problem-solving ability. At the same time, they are satisfied with the DBL approach. These findings suggest that the DBL approach is effective in promoting students' deep learning and improving the quality of teaching and learning.

## Introduction

As global competition intensifies and information technology iterates rapidly, the need for high-quality innovative talent with deep learning ability has become a pressing development need for all countries. Countries around the world have been conducting relevant research through policy traction. In 2012, the National Research Council (NRC) published a study that explored the integration of deep learning with 21st-century competencies. In 2013, Canada promoted the “New Pedagogies for Deep Learning” research project, which aimed to promote the development of core literacy based on deep learning. In 2017, China pointed out in the “Opinions on Deepening the Reform of the Education System and Mechanism” that education reform and development should “focus on cultivating students' key abilities for lifelong learning, innovative thinking and adapting to the requirements of the times”. Exploring teaching strategies for deep learning has become a long-term trend in the development of the world's basic education sector (Zhang, 2015). Promoting the cultivation of students' deep learning ability is an important issue in the development of educational reform (Zhang et al., 2014).

Design-based learning (DBL) is a learning model that has received attention in recent years. It is similar to problem-based learning (Gijselaers, 1996) and problem-based project learning (Chandrasekaran et al., 2013; Kolmos, 2002). DBL emphasizes the learning of scientific knowledge and professional skills through the involvement of learners in designing projects in real-life situations. This approach can increase students' desire to learn and effectively develop learning ability (Doppelt et al., 2008).

In the field of engineering education, DBL's teaching methods are often used to deal with practical projects and design problems to improve the teaching quality of courses. Huang et al. (2020) showed that the application of DBL in engineering education could foster individual sustainability competency. Jiang et al. (2020) used the DBL approach to address the learning challenges of a joint undergraduate course in China and abroad and achieved satisfactory teaching and learning outcomes. Ai et al. (2020) demonstrated that project-based design learning (PODBL) could be effective in improving industrial design teaching, increasing student learning efficiency and enhancing the learning experience. Zhang et al. (2021) combined the DBL approach with an Outcomes-Based Education (OBE) approach to teaching. The results showed that students' competencies were improved. The above-mentioned empirical studies by scholars have achieved some results. However, the development of students' deep learning ability such as knowledge integration ability, problem solving ability and critical thinking ability during the learning process has not been specifically studied.

The key to DBL is to complete the teaching process through design (Huang et al., 2016; Li et al., 2015). As a creative activity, design plays a central role in the innovation process (Hobday et al, 2011). Studies have shown that DBL can improve students' problem analysis ability, innovative thinking ability, critical thinking ability, etc. (Altan et al., 2021; Ding et al., 2011). The improvement of these higher-order thinking and higher-order capabilities is very consistent with the ability requirements of deep learning. Deep learning is a quest for acquiring higher-order competencies, namely critical thinking ability, problem-solving ability and innovation ability, which are essential for innovative talents in the 21st century (Chen & Zhang, 2016). Therefore, promoting deep learning for learners through DBL in the field of engineering design education is a research question that deserves attention and exploration.

In contrast to previous research, this paper focuses on the promotion of deeper learning situations for students through the DBL approach. We have studied the DBL approach in a practical teaching project in schools. In this paper, we explore the following three main research questions.

1. Does the DBL approach promote students' deep learning?
2. Does the DBL approach improve students' academic performance?
3. What are the students' views and comments on the DBL approach?

The paper is organized as follows. The second section reviews the knowledge of the literature on DBL and deep learning. The third section presents a DBL model for promoting deep learning, including materials, procedures and measures for specific implementation. The fourth section outlines the findings of the three research questions. The fifth section discusses the three research questions and compares them with previous research. The sixth section presents the conclusions of the study and directions for further research.

## Background

### Design‑based learning

DBL originated from the “learning by design” model proposed by Kolodner and was later followed by the “design-based scientific inquiry learning cycle” model (Kolodner, 2002). The two main elements of this model are design and redesign, and investigation and inquiry. Later, Nelson created and put into practice and promoted “design-based learning”with considerable success in K12 interactive classrooms (Zhu et al., 2017). Nelson's “Backward Thinking” is one of the well-known DBL learning models. The model is roughly divided into six steps. That is to clarify the theme, determine the problem, establish evaluation standards, establish models, teaching guidance, evaluation and modification. Compared with the traditional teaching model, the “Backward Thinking” model highlights the iterative nature of the learning process and emphasizes the comprehensiveness of various disciplines in the learning challenge.

The “Backward Thinking” model is derived from Bloom's classification of cognitive goals, which classifies cognitive learning from low to high into six levels: knowledge, comprehension, application, analysis, synthesis and evaluation. This is one of the most common ways of evaluating the results of deep learning in terms of cognitive dimensions. Traditional education and learning focus on the memory and understanding of knowledge. However, true learning requires students to actively analyze, synthesize, apply, and evaluate facts and ideas. According to Nelsen, traditional “forward” teaching begins with basic facts, while “Backward Thinking” begins with the highest level of reasoning. Students' higher-order thinking and higher-order capabilities can be developed through the “Backward Thinking” model. In addition, Fortus et al. (2004) proposed the “Design-Based Science learning cycle”. The model consists of five steps: (1) Identify and Define Context; (2) Background Research; (3) Develop Personal and Group Ideas; (4) Construct 2D and 3D Artifacts; (5) Feedback.

From the above model, it can be seen that the DBL approach has the following characteristics. (1) Situational. The DBL approach emphasizes that learners can carry out inquiry activities in a task situation, so that learners can “learn by doing”. (2) Design. This is the core of DBL (Huang et al., 2016; Li et al., 2015). Design is an important tool for problem solving. Design thinking is always present throughout the learning process. (3) Integrative. In the DBL process, students need to participate in small groups and collaborate with others to design. Students tackle design challenges by integrating the knowledge and skills of different disciplines. (4) Iterative. The DBL process is an iterative cycle. Students' design solutions need to be revised and optimized several times. (5) Reflective. Students continue to learn from their experiences through reflection in the design practice process. Teachers provide students with timely feedback, making them clear about their learning and constantly adjust their learning plans.

### Design‑based learning

Deep learning has been a popular research topic of interest in education in recent years. In 1976, Marton and Saljo at the University of Gothenburg introduced deep learning and shallow learning based on differences in the way information was processed (Marton & Saljo, 1976). Deep learning is high level or active cognitive processing, while shallow learning is low-level cognitive processing (Biggs, 1979). Biggs (1999) argued that deep learning was the process of making connections between old and new knowledge through critical thinking. Entwistle (2000) argued that deep learning was an active way of learning with the aim of understanding meaning. Zhang et al. (2012) argued that deep learning advocated active, critical and meaningful learning. From the above discussion, it is clear that deep learning refers to learners critically learning and reflecting in authentic situations, actively making connections between old and new knowledge systems, and solving complex problems through deep information processing.

Currently, research on deep learning has been conducted in three main types. In the first type, deep learning is used as a learning approach. Wang et al. (2021) applied deep learning theory to instructional design, and the results showed that the new teaching approach achieved high satisfaction levels, as well as improved student performance. He et al. (2021) combined deep learning with college ideological and political courses to teach. The results showed that the improved curriculum was able to attract students and led to progress in terms of values, ideology and knowledge base. In the second type, deep learning is used as a learning process. Among these, the 3P (Presage-Process-Product) model, developed by Biggs, has been influential. This model measures deep learning in terms of two dimensions: motivation and strategy. In the third type, deep learning is used as a learning outcome. By exploring effective teaching models to promote deep learning among students. Chen et al. (2016) designed a flipped classroom that led to an increase in graduate students' cognitive level and significantly enhanced deep motivation and engagement in learning. Terrenghi et al. (2019) investigated in a high school classroom that “Episode of Situated Learning” in a high school classroom and showed that it could increase student engagement and promote deep learning.

Although scholars have conducted research in classrooms across different disciplines, there is less theoretical and practical research on the assessment of students' deep learning. The National Research Council (NRC) has classified the competencies developed by learners in deep learning into three main dimensions: cognitive, interpersonal and personal (Trigwell & Prosser, 2001). This is highly consistent with the six competencies for deep learning proposed by the Hewlett Foundation (Pu et al., 2016). As shown in Table 1, the framework breaks away from traditional cognitive boundaries to include the interpersonal and self domains in the evaluation of deep learning. It provides an important reference for constructing specific evaluation dimensions and metrics.

**Table 1:** Deep learning ability framework incorporating Hewlett Foundation and NRC

NCR Deep Learning Dimension	Hewlett Foundation 21st Century Competencies
Cognition	Critical thinking
	Problem solving
Interpersonal	Team cooperation
	Effective communication
Personal	Learning to learn
	Learning Perseverance

### Design-based learning and deep learning

The DBL approach emphasizes responding to challenges, integrating knowledge and applying skills to solution design in real-life situations. Students use established evaluation criteria to continually optimize their schemes, proactively add the required knowledge and ultimately achieve a problem-solving outcome. The approach is in line with Bloom's classification of cognitive goals. Learners achieve the cognitive level by applying knowledge, analyzing problems, synthesizing solutions and evaluating designs. At the same time, DBL also corresponds to the highest level of learning in Gagne's hierarchy system of learning. It means that learning takes place through problem solving. He et al. (2005) identified problem-based learning, task-based learning and process assessment as teaching strategies that effectively promoted deep learning. In 2015, the Horizon Report Basic Education Edition also identified project-based learning, problem-based learning, inquiry-based learning, and challenge-based learning as deep learning approaches that help students gain more active learning experiences (Jiao, 2015). Students learn more deeply when they are asked to design and produce work that requires understanding and application of knowledge (Feng & Miao, 2009). In the process of solving design problems, DBL enables learners to master the core content of the subject and apply their knowledge. It is a deeper approach to learning that enables individual knowledge construction (Zhu et al., 2017) and promotes the integration of multidisciplinary knowledge (Puente et al., 2011).

Many scholars have studied DBL to enhance learners from the empirical level. Kafai (2006) used DBL in teaching game programming, and the investigation showed that students' knowledge integration ability as well as their expressive ability were improved. Rao et al. (2018) studied that DBL could effectively promote students' creativity development. In a “Bridging Program” project conducted by Ayub et al. (2015), DBL greatly improved students' critical thinking ability.

In general, DBL incorporates the concept of deep learning to a certain extent (Du et al., 2013). The method of DBL can effectively exercise higher-order thinking and higher-order capabilities and promote deep learning among learners. As the future development trend of education and learning concepts, deep learning is an important goal pursued by DBL.

## Method

### Teaching model of DBL for deep learning

**Fig. 1:**
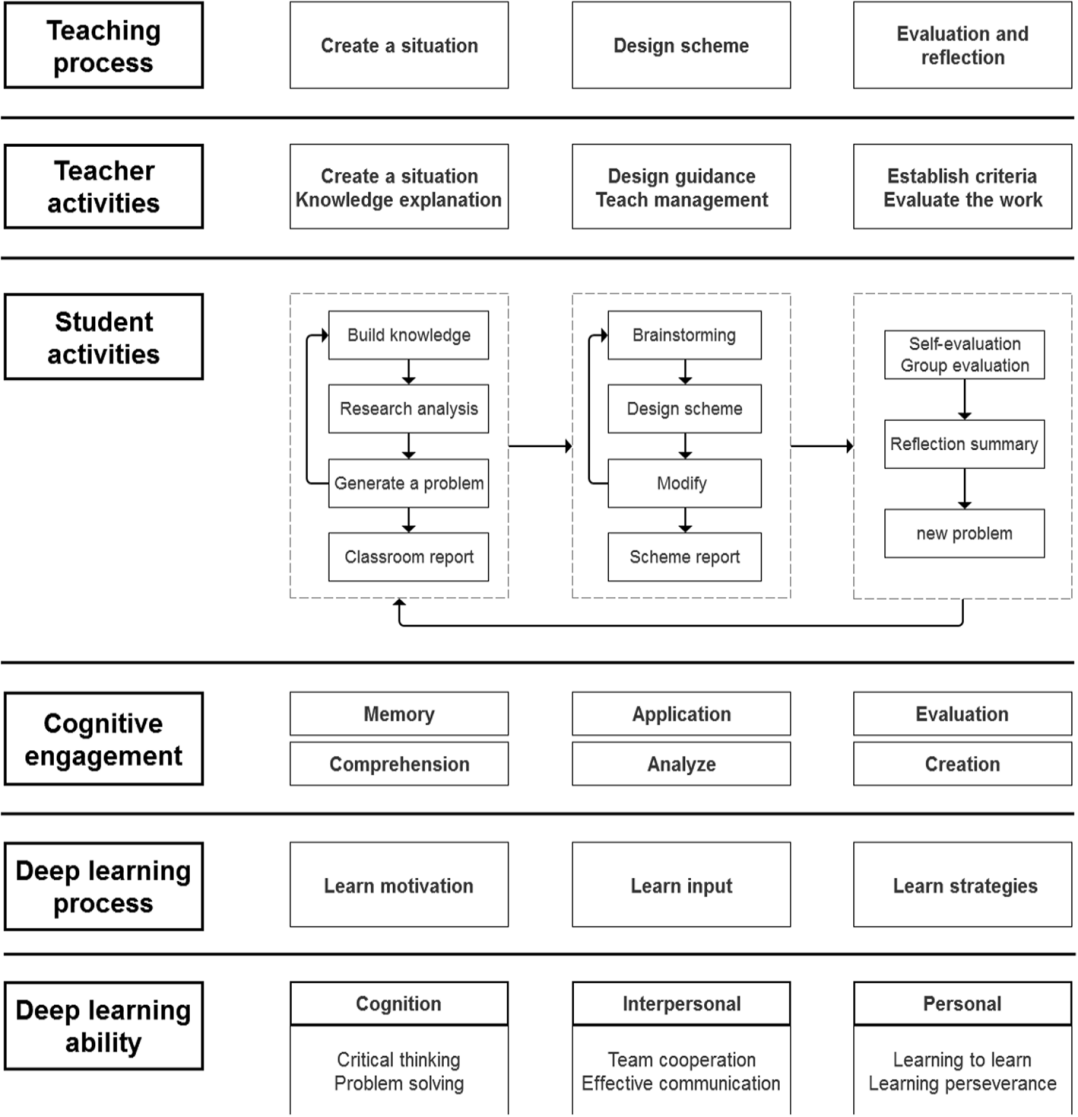
Teaching model of DBL for deep learning

According to the learning model and characteristics of DBL, and combined with the actual situation of engineering design education, this paper constructs a DBL teaching model that promotes deep learning from the perspectives of teaching stages, teacher and student activities and deep learning. As shown in Fig. 1, it is divided into three stages in total. These include the stage of creating a situation, the stage of designing scheme, and the stage of evaluating and reflection.

### Creating a situation stage

The activities of the teacher are arranged as follows. In the first step, the teacher carefully arranges the teaching and learning tasks according to the teaching objectives and the actual situation. The learning task needs to be challenging and stimulate the students' willingness to participate and investigate. In the second step, the teacher creates a teaching situation based on the design of the task so that the content can be taught. It includes teaching the necessary new knowledge and skills. In the third step, the teacher groups students and provides the necessary resources and technical tools for the task.

The students' activities are arranged as follows. In the first step, students memorize and understand the new knowledge explained by the teacher and actively recall their previous knowledge base. In the learning process, the old and new knowledge systems are constantly coupled and deepened. In the second step, students work in small groups to identify what problems and constraints exist through information gathering and analysis tasks. Students serve a common practical project by integrating and sharing information and resources from different areas of expertise. In the third step, students collate and analyze the issues from their research, including core and secondary issues, and share them in class using a PowerPoint presentation.

At this stage, students are fully motivated to learn. Through the memorization and understanding of theoretical knowledge in the research process, their ability to integrate and analyze information resources is exercised. It facilitates the formation of a systematic framework for compiling knowledge. At the same time, students learn to analyze problems critically and prioritize them.

### Design scheme stage

The teacher's activities are arranged as follows. In the first step, the teacher assists students in interpreting the problem and finding practical problem points. In the second step, the teacher communicates the students' initial design scheme and determines the general design objectives and directions. In the third step, the teacher manages and organizes the students' instruction and asks them to plan their time.

The students' activities are arranged as follows. In the first step, students brainstorm through discussions between groups. Each person designs a considerable number of schemes. In the second step, students communicate within the group based on their schemes and select the appropriate ones. In the third step, students revise and optimize their chosen schemes. Later, a presentation is made and shared in class.

At this stage, students deepen their impressions through the application and analysis of their knowledge. At the same time, students learn to communicate with each other and solve problems together. Students have a broader design thinking, a richer design language and a more comprehensive approach to design.

### Evaluation and reflection stage

The activities for teachers are arranged as follows. In the first step, the teacher designs evaluation criteria of the work based on the course content. In the second step, the teacher marks the students' work according to the evaluation criteria and gives suggestions for revision.

The students' activities are arranged as follows. In the first step, students will display and present their designs. In the second step, students self-evaluate their work and evaluate each other in groups according to the evaluation criteria. In the third step, students make timely revisions and reflections based on the feedback and comments from the assessment.

At this stage, students optimize and iterate on the design scheme based on actual situations and summaries of comments. They learn to reflect, acquire learning methods and skills, and persevere for their learning goals.

### Teaching Case

#### Course design and implementation

This study was carried out in the postgraduate course “Artistic Design and Methodology” at the Wuhan University of Science and Technology, School of Art and Design. The teaching team consisted of a lead teacher and two assistant teachers. The total duration of the course was 8 weeks. It consists of 32 class hours. The stage of creating a situation took 12 class hours. The stage of design scheme took 12 class hours. The stage of evaluation and reflection took 8 class hours. The course was designed according to the school's situation and teaching needs. A project theme was identified. It was “Library Service Design for Wuhan University of Science and Technology”. The project met three criteria. Firstly, it was based on actual projects in schools and districts. It allowed students to conduct research conveniently. Secondly, it met the teaching needs of students from different disciplines. Students' design thinking and design skills were effectively exercised. Thirdly, it allowed students to think and design critically.

Responses from school librarians indicated a high level of student occupancy in the study rooms, especially during exam weeks at the end of the semester. Teachers said that the specialist books in the library did not meet demand. Students said that the library's infrastructure was inadequate. For example, e-book resources were not abundant. Some students thought that the library was currently singular in its functions. For example, the supporting facilities were not good enough and the service system needed to be improved. In response to these phenomena, students of different design disciplines needed to play their respective professional characteristics to deal with them together.

**Fig. 2:**
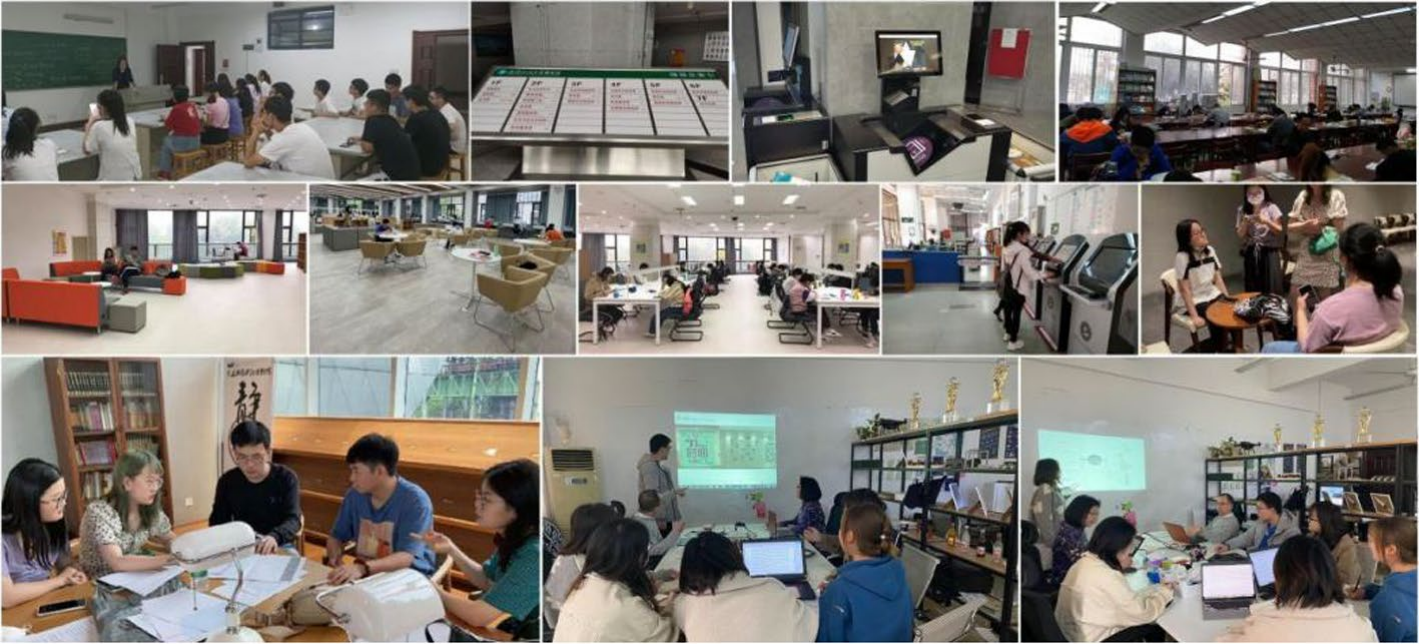
DBL course: library service design based on the real-life scenario

Thus, the design problem: the subject combines the characteristics of each profession to design a solution that will improve the current service situation of the library and provide a better service to the school staff and students. This problem involves many areas of knowledge, including space planning and design, guidance system design and human-machine interaction design, etc. Figure 2 shows a picture of the DBL course.

In the creation of situations stage, teaching focused on theoretical knowledge and case studies. This stage was based on design methods and project research and focused on developing knowledge integration and problem analysis abilities.

Firstly, students were given a focus on the theoretical knowledge of the five stages of design thinking, including knowledge of empathy, knowledge of definition, knowledge of ideation, knowledge of prototyping and knowledge of testing. It was also complemented by user experience theory. This consists of five levels: strategy, scope, structure, skeleton, and surface. The different bodies of expertise that students received at the undergraduate level resulted in different levels of acceptance and understanding of new knowledge. Some students started with relatively small design entry points, resulting in a poor understanding of the “strategic layer” and confusion between the “structural layer” and the “skeleton layer”. Some students also had a poor understanding of the “defining knowledge” and did not know how to use design methods to define problems. As barriers to knowledge learning were exposed, this allowed knowledge from different design disciplines to collide and a couple in students' minds.

Secondly, the students began to research and gather information about the library using design methods. This included environmental information, user information, process information, etc. Environmental information included the size of the site, the distribution map of the functional areas, the condition of the services and the information guidance system. User information was gathered through questionnaires and user interviews. The students conducted a comprehensive analysis of the user group, explored their pain points and needs, and also drew a user profile. Process information was presented through user experience maps and service blueprints. The team consolidated all the information about the project, which was comprehensively integrated and analyzed.

**Fig. 3:**
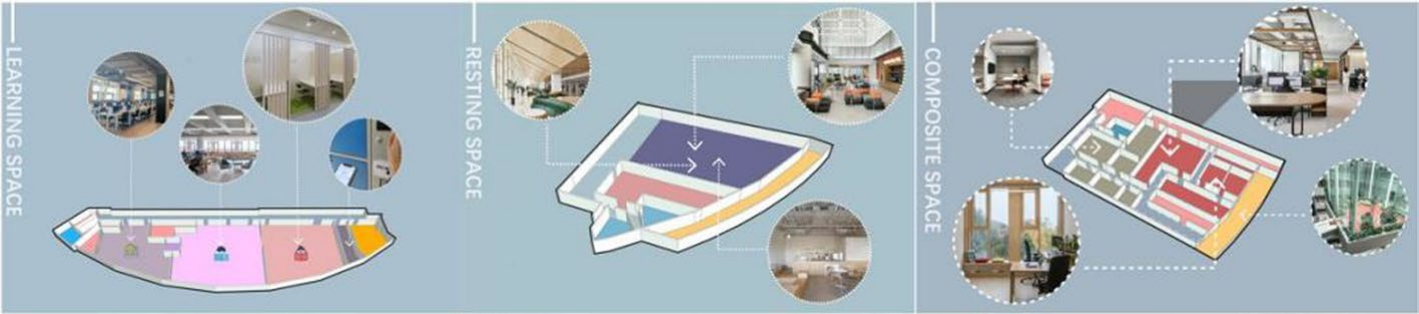
Some of the students' design schemes

In the design scheme stage, teaching focused on critical thinking and design skills. This stage focused on developing students' teamwork and effective communication abilities. Students collated and analyzed the problems from their research. Each group prioritized the issues that needed to be addressed, including core and secondary issues. Students began to look for relevant and good examples of design highlights to learn from. Secondly, each group communicated with the teacher on time about the appropriateness of the overall design idea. The group needed to define the design objectives to combine the design opportunity points with the benefit points. The group members consulted with each other several times to complete the scheme. Took one group of students as an example. They modified the overall space on the second level without changing the essential functions. Figure 3 shows the students' initial design ideas. In the study space, the phenomenon of seat occupancy improved by dividing reserved study areas and non-reserved study areas. In the resting space, the cafe was designed not only to meet the needs of candidates for a short break but also to help relieve the pressure of studying. In the composite space, the meeting area on the second level of the library was reduced and a discussion area was added. At the same time, they transformed an under-utilized space into a recitation area. It met the recitation needs of students while avoiding disturbing other students who were studying.

In the evaluation and reflection stage, the teaching focused on guiding students in self-evaluation and peer evaluation. This stage promoted mutual learning and competition and triggered self-reflection. The focus was on developing students' learning ability and endurance. Afterwards, the teachers held a presentation of the results. Members of each group explained their design schemes and design ideas. The teacher and other group members asked questions to prompt changes and design iterations. Students also learned about different design ideas and schemes from this.

### Participants

The teaching of the course took place over two years, including 2019 and 2021 (the online course conducted in 2020 due to COVID-19 did not allow for a situational teaching session). A total of 49 postgraduate students participated in the course in the class of 2019. The class was taught using traditional design instruction and set as a control class. A total of 56 postgraduate students participated in the course in 2021. The class was taught using DBL teaching and set as an experimental class. They all came from different design disciplines and had a good knowledge base. The experimental class included 16 product design students, 14 environmental design students, 21 visual communication design students and 5 public art design students. Before the course started, 56 students were divided into 7 groups. Each group consisted of 8 students, 1 group leader and 7 group members. There were at least 3 different majors in the group to fully ensure that the groups were well staffed. The same grouping was used for the control class. At the end of the course the experimental and control classes used the same marking criteria to mark the students' design work.

### Evaluation criteria

At the end of the course, each student scored based on teacher and student evaluations and student performance in class. The specific evaluation criteria were listed in Table 2. The results were divided into four grades based on the four dimensions of integrity, rationality, innovativeness and aesthetic, grade A (100-90), grade B (89-80), grade C (79-70) and grade D (69-0). The total score was 100, with 50 points for integrity, 20 points for rationality, 20 points for innovativeness and 10 points for aesthetic. Integrity means that the work was very complete, with rich content and good professional division of labour. Rationality represented a work that was realistic and highly feasible to operate. Innovativeness represented work that solved practical problems and created good value. Aesthetic represented work that reflected good design skills and aesthetic ability. The specific calculation of each student's grade is as follows: Y = x1 × 60% + x2 × 20% + x3 × 10% + x4 × 10%. Y represented the total student achievement score, x1 represented the teacher assessment score, x2 represented the group mutual assessment score, x3 represented the individual self-assessment score, x4 represented the classroom performance score and attendance score. Students and teachers graded on the basis of uniform evaluation criteria. Ultimately, the student's course grade was based on the percentage of marks given by teachers and students.

**Table 2:** Criteria for evaluating the performance of work

Grade	Points	Integrity (50 Points)	Rationality (20 Points)	Innovativeness (20 Points)	Aesthetic (10 Points)
A	100–90	The integrity of the work is very good, with a very good professional team division of labour	The work is highly feasible in practice and follows a sound logic	The innovation of the work is very good	The work reflects very good design skills and aesthetic ability
B	89–80	The integrity of the work is good, with a good professional team division of labour	The practical feasibility of the work is good and follows a sound logic	The innovation of the work is good	The work reflects good design skills and has relatively good aesthetic ability
C	79–70	Relatively good integrity of work, with a good division of work between professional teams	The practical feasibility of the work is relatively good, following a sound logic	The innovation of the work is relatively good	Works reflect relatively good design skills and aesthetic ability
D	69–0	Lack of integrity in the work and lack of good professional teamwork	Lack of practical feasibility of the work and lack of sound logic	The innovation of the work is not enough	The work reflects the lack of design skills and aesthetic ability

### Research instruments

This study was based on the “deep learning: opportunities and outcomes (student questionnaire)” developed by the deep learning research project SDL, on Biggs' learning process scale (SPQ) and the deep learning questionnaire developed by Li et al (2018). The Deep Learning Process Questionnaire (Appendix 1) and the Deep Learning Ability Questionnaire (Appendix 2) were designed. Both questionnaires were scored on a 5-point Likert scale, ranging from “strongly disagree”, “disagree”, “not necessarily”, “agree” to “strongly agree” on a scale of 1, 2, 3, 4 and 5, respectively.

### Data collection

#### Questionnaires

A deep learning process questionnaire and a deep learning ability questionnaire were asked to be completed by each of the 56 students in the experimental class before and after the course. The 56 questionnaires were returned respectively, with a 100% return rate. The paper also collected the perceptions of the 56 students in the experimental class about DBL teaching from six dimensions.

#### Student's grade

A comparative analysis of the performance of 56 students in the experimental class and 49 students in the control class based on the same assessment criteria was carried out after the lesson. The discussion focused on the distribution of student achievement.

#### Depth interviews

After the lesson, one student from each of the different levels of achievement scores in the experimental class was selected for depth interview. This was to gain a full understanding of the learning experience of students with different grades.

## Results

### Questionnaire test

In order to ensure the reliability and validity of the “deep learning process questionnaire” and “deep learning ability questionnaire”, two questionnaires were distributed to 78 students before formal teaching. In this study, the questionnaire was analyzed for reliability and validity using IBM SPSS 25.0, as shown in Tables 3 and 4. The deep learning process questionnaire consisted of three dimensions: learning motivation (A1–A5), learning input (A6–A11) and learning strategy (A12–A16). There were five questions in the “learning motivation” dimension, six questions in the “learning input” dimension and five questions in the “learning strategy” dimension. The overall reliability coefficient of the Deep Learning Process questionnaire was 0.869, indicating that the overall reliability of the questionnaire was very high, and the KMO measure was 0.866, meaning that the validity of the questionnaire was good. The deep learning ability questionnaire consisted of three first-level dimensions: cognitive, interpersonal and personal. It also consisted of six second-level dimensions: critical thinking (B1–B3), problem solving (B4–B6), teamwork (B7–B9), effective communication (B10–B12), learning to learn (B13–B15) and learning to persevere (B16–B17). There were six questions in the “cognitive” dimension, six in the “interpersonal” dimension and five in the “personal” dimension. The overall reliability coefficient of the deep learning ability questionnaire was 0.888, and the reliability coefficients of the other primary and secondary dimensions were all above 0.7. The KMO value of 0.843 indicated that the questionnaire had good validity.

**Table 3:** Deep learning questionnaire reliability data

Questionnaire	Dimension	Number of items	Cronbach’s Alpha
Deep Learning Process Questionnaire	Learning motivation	5	0.885
	Learning input	6	0.908
	Learning strategy	5	0.936
	Overall	16	0.869
Deep Learning Ability Questionnaire	Cognition	6	0.901
	Interpersonal	6	0.917
	Personal	5	0.906
	Overall	17	0.888

**Table 4:** Deep learning questionnaire validity data

Questionnaire	Sig	df	Approximate chi-square	KMO
Deep Learning Process Questionnaire	0.000	120	916.868	0.866
Deep Learning Ability Questionnaire	0.000	136	985.484	0.843

### Deep learning status results

As shown in Table 5, the experimental classes differed significantly (*p* < 0.05) in the dimensions of learning motivation, learning input and learning strategy. In the pre-test data, the mean values for the three dimensions of students' deep learning status were 3.46, 3.67 and 3.41. After the implementation of DBL instruction, all three were improved, with mean values of 4.03, 3.97 and 3.74 for the post-test data. Learning motivation increased relatively the most before and after the implementation of DBL. The mean score of 4.03 was relatively high. This indicated that students' learning motivation increased and they were more inclined to take the initiative in learning during the teaching and learning process.

**Table 5:** Deep learning process questionnaire results

Dimension	Pre-test		Post-test		Number of items	T	*P*
	Mean	SD	Mean	SD			
Learning motivation	3.46	0.48	4.03	0.56	56	−7.906	0.000
Learning input	3.67	0.51	3.97	0.71	56	−3.546	0.001
Learning strategy	3.41	0.49	3.74	0.65	56	−3.065	0.003

### Deep learning ability results

The results in Table 6 shown that the *p* values for the post-test were all less than 0.05, indicating that there were significant differences between the experimental group's six deep learning abilities before and after the experiment. All abilities improved compared to the previous ones. Among them were critical thinking ability (T=−3.173, *p *= 0.002 < 0.05), problem solving ability (T=−3.830, *p *= 0.000 < 0.05), and learning to learn ability (T=−3.923, *p *= 0.000 < 0.05). This indicated that students' improvement in the three dimensions of critical thinking, problem solving and learning to learn was particularly evident. In the interpersonal area, there was a significant difference between the pre-test and post-test results for effective communication ability (*p *= 0.0026 < 0.05). It indicated an increase in effective communication ability. However, the mean values of 3.22 and 3.60 for the pre-test and post-tests were relatively low. It indicated that there was a need to further improve teaching strategy in the study of effective communication.

**Table 6:** Deep learning ability questionnaire results

Dimension	Deep learning ability	Pre-test		Post-test		Number of items	T	*P*
		Mean	SD	Mean	SD			
Cognition	Critical thinking	3.34	0.76	3.80	0.83	56	−3.173	0.002
	Problem solving	3.24	0.75	3.87	0.82	56	−3.830	0.000
Interpersonal	team cooperation	3.42	0.66	3.74	0.66	56	−2.715	0.009
	Effective communication	3.22	0.87	3.60	0.75	56	−2.292	0.026
Personal	Learning to learn	3.30	0.69	3.82	0.61	56	−3.923	0.000
	Learning perseverance	3.40	0.78	3.75	0.67	56	−2.619	0.011

### Student grade results

The results of the control and experimental classes were graded separately after the lesson according to the same grading scale. As Table 7 shown, the experimental class performed better than the control class after the optimization of the teaching reform. Their difference reached a significant level (t = 3.561, df = 103, *P*< 0.05).

**Table 7:** Results of the control and experimental class

Class type	N	Mean	t	df	*P*
Control class	49	82.674	3.561*	103	0.001
Experimental class	56	86.571			

*p < 0.05

**Fig. 4:**
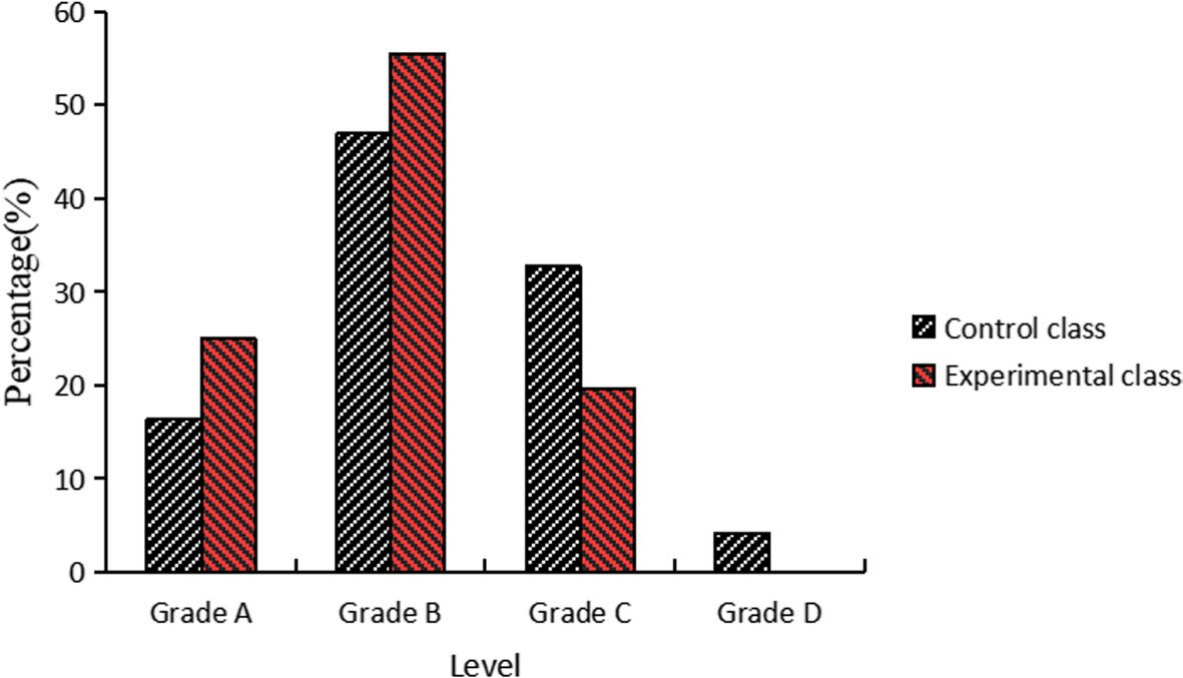
Student grade comparison results

As shown in Figure 4, the percentage of students in the control class scoring in the four grades of A, B, C and D were 16.3%, 46.9%, 32.7% and 4.1% respectively. The experimental class scored 25.0%, 55.4%, 19.6% and 0.0% in the four grades A, B, C and D respectively. After the implemented DBL teaching model, the experimental class scored significantly better at A and B levels, significantly lower at C level and had zero D level scores. It indicated that the reformed curriculum had led to an increase in the proportion of students achieving outstanding results. The situation of students' design thinking and design skills has improved. It indicated that the DBL teaching model was effective in improving the quality of teaching and learning.

### Teaching evaluation results

To measure students' perceptions of DBL teaching, Table 8 showed the survey results based on the Likert scale. The combination of “strongly agree” and “agree” combined creates a positive answer of “agree”. Similarly, “disagree” and “strongly disagree” combined form a negative answer of “disagree”. “Not necessarily” was a neutral attitude. As shown in Table 8, 85.7% of the responses were positive in terms of “active and deep learning”. Only 3.6% were negative. It indicated that active thinking and deep learning could be effectively promoted by changing the curriculum. The second highest value of 82.7% of positive responses was “innovative and interesting teaching”. It had only 5.4% negative responses. Only 69.7% of the positive responses were “rich and efficient interaction”. There was also a neutral response of 19.6% and a negative response of 10.7%. It indicated that interaction between students from different majors was problematic and that there was a need for more logical ways to bring students closer together. The answers for “scientific and rational training”, “diverse and integrated knowledge” and “timely and rapid feedback” were all highly positive, at 73.2, 76.7 and 74.9% respectively. In summary, the students accepted the new teaching model. At the same time, the students were pleased with the content and effectiveness of the instruction. In terms of teaching organization, the communication and interaction within the different groups was not good enough, which needed further improvement later on.

**Table 8:** Students' evaluation results of DBL teaching

Variables	SD (1) (%)	D (2) (%)	Total (1 + 2) (%)	N (3) (%)	A (4) (%)	SA (5) (%)	Total (4 + 5) (%)
Scientific and rational training	5.4	7.1	12.5	14.3	39.3	33.9	73.2
Diverse and integrated knowledge	3.6	3.6	7.2	16.1	46.4	30.3	76.7
Timely and fast feedback	5.4	5.4	10.8	14.3	44.6	30.3	74.9
Rich and efficient interaction	3.6	7.1	10.7	19.6	41.1	28.6	69.7
Active and deep learning	1.8	1.8	3.6	10.7	44.6	41.1	85.7
Innovative and interesting teaching	3.6	1.8	5.4	12.5	42.8	39.3	82.1

### Depth interview results

One student from each of the experimental classes with an A, B and C grade was selected for an interview after the lesson (Appendix 3). The results of the interviews were presented below. An A-level student found the course activities meaningful. He would take the initiative to learn different professional knowledge and look at issues critically. He felt a little different when interacting with students from different majors but was able to deal with it on time. A B-level student found the course very practical as they learned to apply their knowledge to solve practical problems. The course content was challenging. However, through their learning and working with students from different majors. The student solved the problems well and gained a sense of achievement. A C-level student felt that the course required collaboration with students from different majors. We all had different perspectives on understanding the problems, which led to some barriers to collaboration. However, they took the initiative to learn and contribute to the group. It was clear from the above results that students with good grades took the initiative to construct their own body of knowledge. They could deal critically with problems and communicate efficiently with students from different majors. The low scorers were still confined to their expertise and did not take the initiative to learn different professional content. They were narrowly considered and mechanical in their approach to problems.

## Discussion

In this paper, the DBL method was applied to the teaching of graduate students majoring in engineering design in colleges and universities. It focused on the deep learning and teaching situation of students. Based on the findings of the research, the three questions raised in the introduction section are discussed.

### Does the DBL approach promote students' deep learning?

The results of the questionnaire indicated that the deep learning of the students had improved. In terms of deep learning status, students' learning motivation was higher. They were more engaged in their learning, and their learning strategy were strengthened. In terms of deep learning results, students' critical thinking ability, problem solving ability, and learning ability had improved significantly. At the same time, teamwork ability and learning perseverance had improved. For effective communication, the improvement was relatively low. It required further research later. Over, the new teaching model helped develop higher-order thinking and higher-order capabilities and promoted deep learning for students.

### Does the DBL approach improve students' academic performance?

The survey results indicated that students' grades had improved. The percentage of exceptional students had risen. Student achievement evaluation criteria are an important aspect of determining the quality of teaching and learning. This paper construed specific evaluation criteria. Students and teachers marked the work according to the evaluation criteria. The results showed a significant improvement in student performance through the teaching reform. The percentage of students who excelled increased more significantly. It indicated that the new teaching method had improved the quality of teaching and learning and helped to foster better talents.

### What are the students' views and comments on the DBL approach?

The survey results indicated that students were more satisfied with the DBL method. They felt that it was a novel way of teaching. The interactive atmosphere of the classroom was increased through practical projects. It broke with the traditional rigid indoctrination style of teaching. They felt that the DBL method helped to promote their active thinking and deep learning.

In contrast to previous research, this paper focused on the deep learning and teaching situation of students. Many researchers also discussed the application of DBL in specific teaching situations. They studied the teaching effectiveness of the DBL approach (Jiang et al., 2020). They focused on students' learning experiences (Ai et al., 2020). Some scholars studied the implementation of DBL for cultivating students' ability (Huang et al., 2020; Puente et al., 2011). Some scholars conducted empirical studies combining the DBL method with other teaching methods (Zhang et al., 2021). Zhang et al. (2022) studied that specific DBL activities affect students' emotional experiences.

However, the above studies were not specific about the deep investigation of students' thinking and ability. The application and implementation of teaching methods should focus on the long-term development of students and the development of deeper thinking and ability. This paper focused on the specific situation and teaching of DBL for promoting students' deep learning. The results indicated that the DBL method could effectively promote students' deep learning and improve the quality of teaching and learning.

## Conclusions

This paper examines the impact of the DBL model of teaching on students' deep learning profiles. The correlation between DBL and deep learning is understood through a review of the relevant literature. A DBL teaching model to promote deep learning and criteria for evaluating student performance have also been constructed. The deep learning questionnaires and the study of students' performance indicate that students have increased motivation and are more inclined to take the initiative in their learning. Students' critical thinking ability and problem solving ability have been significantly improved. At the same time, there is a significant increase in the proportion of students achieving outstanding grades. It demonstrates the effectiveness of the DBL model in promoting deeper learning and improving the quality of teaching and learning.

This research has several limitations that will need to be addressed in future research. Firstly, the sample size of this study is small and limited to a specific teaching context. The applicability to other areas of engineering education requires further research. Secondly, this research does not go far enough into the investigation of deep learning and lacks more authoritative and comprehensive research. Finally, this research lacks an authoritative survey of student learning satisfaction. All these issues need to be further researched in future teaching. The deep learning situation of students is better facilitated through improved teaching methods. To better determine the effectiveness of teaching methods, this paper will investigate a wider range of implementations.

## Appendix 1: Deep learning process questionnaire

**Table:**

Dimension	Questions
Learning motivation	A1. Will learning give me a deep sense of personal satisfaction?
	A2. I feel very happy and satisfied when learning new things?
	A3. Learning is a very rewarding and meaningful thing to do?
	A4. I find that sometimes researching design issues is as exciting as reading a good novel?
	A5. The purpose of learning is to learn to think and to acquire knowledge?
Learning input	A6. I will devote a lot of time to learning activities?
	A7. I forget everything around me when I'm studying?
	A8. I am always able to maintain my concentration when browsing online learning resources?
	A9. I will use different approaches to solve design problems?
	A10. When learning new things I relate them to previous knowledge?
	A11. I am interested in design activities?
Learning strategy	A12. I can get the information I want from a variety of sources?
	A13. I usually make detailed changes to the design plan?
	A14. I often discuss design with my teacher?
	A15. I would accept understanding the different design perspectives?
	A16. In the class, I will seriously participate in the classroom activities and think positively?

## Appendix 2: Deep learning ability questionnaire

**Table:**

Dimension	Questions
Cognition	B1. I always analyze the focus of a problem before solving it?
	B2. I am good at developing a systematic plan to solve complex problems?
	B3. I think rationally about different design ideas?
	B4. I always try to solve problems by myself?
	B5. I will break down complex problems and solve them?
	B6. I will use subject area knowledge, strategies and tools to solve problems?
Interpersonal	B7. I can help the group to complete tasks and solve problems?
	B8. I enjoy working with my group members?
	B9. I feel that teamwork is more powerful?
	B10. I will share my views and ideas in the group?
	B11. Group members give timely feedback on my ideas?
	B12. I am able to generate new ideas through group work and communication?
Personal	B13. I will make a plan to get things done?
	B14. I will try to get help to achieve my goals?
	B15. I can find the materials and information I need for my studies?
	B16. I can overcome the difficulties to achieve my goals?
	B17. I will persevere even if it takes a long time to complete the task?
